# The needs of family caregivers providing palliative home care in Portugal: a multi-stage mixed methods study protocol

**DOI:** 10.3389/fpubh.2025.1596657

**Published:** 2025-06-02

**Authors:** Carlos Laranjeira, Maria dos Anjos Dixe, Alexandra Coelho, Carla Reigada, Rui Carneiro, Ana Querido

**Affiliations:** ^1^School of Health Sciences, Polytechnic University of Leiria, Leiria, Portugal; ^2^Centre for Innovative Care and Health Technology (ciTechCare), Polytechnic University of Leiria, Leiria, Portugal; ^3^Comprehensive Health Research Centre (CHRC), University of Évora, Évora, Portugal; ^4^Applied Psychology Research Centre Capabilities & Inclusion, ISPA – Universitary Institute, Lisbon, Portugal; ^5^Universidad Internacional La Rioja, La Rioja, Spain; ^6^Universidad El Bosque, Bogotá, Colombia; ^7^Internal Medicine and Intensive Care Department, Hospital da Luz – Arrábida, Vila Nova de Gaia, Portugal; ^8^RISE-Health, Nursing School of Porto (ESEP), Porto, Portugal

**Keywords:** family caregivers, palliative care, needs assessment, advocacy, community support, mixed methods, Portugal

## Abstract

**Background:**

In palliative care (PC), family caregivers (FCs) play an important role in managing patient symptoms and addressing patient needs. In end-of-life (EoL), FCs frequently experience distress that exacerbates emotional strain and complicates grieving. Training FCs to care for palliative patients should be implemented urgently, enhancing their preparation, reducing their burden, and assuring Quality of Life (QoL) throughout illness progression. Recent research has highlighted a global shift toward death in the community, in line with patient preferences. In contrast, the Portuguese reality reveals a tendency to die in hospitals and an absence of community PC and support for FCs, a model that might not be sustainable in the future.

**Aims:**

The overall aim of this study is to comprehensively assess the unmet needs of FCs in home-based PC settings and their experiences interacting with PC services, and to propose strategies and recommendations for FC advocacy in PC.

**Methods:**

A multi-stage mixed-methods design will be used, divided into four main phases. Phase I will identify unmet needs and profile FCs through a quantitative cross-sectional analysis of a nationally representative sample. Phase II will develop a qualitative study to understand the role and impact of FCs providing PC and their experiences with support from PC services. This will help generate ideas for more accessible and sustainable PC-in-place. Phase III will comprise a multi-phased, consensus-based approach to identify priority areas of need, as decided by FCs and professionals, and develop a short Caregivers Assessment Tool (CAT). Lastly, phase IV will synthesize the results and produce a white book for FC advocacy in PC.

**Discussion:**

The project will enrich community PC while optimizing social welfare activities. By identifying the unmet requirements of FCs of PC patients, the initiative will enhance the QoL and well-being of the care recipients, respecting their preferences, while improving the health and competence of FCs, and minimizing the consumption of hospital resources. Lastly, FC engagement should be coordinated and sustainably executed through the participation of relevant all stakeholders.

## 1 Introduction

In Portugal, as in other European nations, palliative care (PC) is acknowledged as a human right. The Basic Law for Palliative Care (Law No. 52/2012) established the National Network for Palliative Care (NNCP) to deliver active and comprehensive support to patients with severe ill conditions and their families. The NNCO encompasses several specialized PC units/teams, in hospitals, the community, or at home (1, 2).

There has been a recent uptick in providing PC patients with in-home care services, perhaps driven by the rising number of people who prefer being cared for and dying in the comfort of their own home rather than in a hospital or nursing home (3, 4). Evidence demonstrates that home death is unlikely without family caregivers (FCs) (5), highlighting their importance in enabling end-of-life (EoL) care at home. Promoting home-based care is economically advantageous for healthcare systems, decreasing emergency room visits and hospitalizations, while alleviating inequities in access to PC (1, 2). Nonetheless, the progress of home-based PC necessitates the fulfillment of specific criteria. There must be a family member capable of assisting the patient in the absence of social and healthcare professionals tasked with home-based care. Therefore, the implementation of home-based PC programs might be difficult, particularly in cases involving individuals who reside alone or together with older people (1, 2). Alongside the challenges posed by families’ social contexts, systemic deficiencies within the healthcare framework, including resource limitations and shortages in professional training—especially in generalist PC—impede the evolution of this practice, despite the existence of established legal rights.

Caring for someone at the EoL can be arduous and demanding: one in ten FCs attending to a patient at EoL encounters a care-related burden (6–8). In a home environment, this burden can be especially significant, as the patient increasingly relies on FC assistance. Additionally, FCs may suffer anticipatory grief of their relative’s approaching death (6, 9). Among at-home FCs, the probability of significant burden rises from 32% in the second and third months prior to death to 66% one week before death (10). Burden may lead to physical and psychological morbidity, limitations on the caregiver’s life, and a burden on financial resources (11–13). Older individuals relying on family caregivers are particularly vulnerable to physical and psychological health complications during EoL care (14, 15). The COVID-19 pandemic underscored the need to acknowledge frontline FCs (16). FCs must secure timely help to avert overload and improve their caregiving capacity. This may also pertain to the quality of life and, eventually, the quality of the patient’s final days. Healthcare providers possess multiple methods to assist FCs. Generally, these can be categorized into two domains: (1) empowering FCs in delivering care (as “co-workers”) (17), which includes practical aid with caregiving tasks, care coordination, information provision (18–21), and support in symptom management or medication administration (18, 22, 23); and (2) providing psychosocial support designed to enhance the wellbeing of the FC (as “co-client”) (13), encompassing respite care (e.g., day-care programs for temporary relief) (24), emotional support, and addressing social needs (18–21).

Research has identified several primary support needs, including increased time off, clear expectations for the future, practical assistance at home, health-related support, and guidance in managing emotions and concerns (8, 25). Many of these support needs involve providing the FCs with specific assistance rather than support aimed at enhancing their caregiving capacities, suggesting that FCs should be seen as co-clients and care partners (25). Nevertheless, healthcare practitioners typically adopt a more client-centered approach, prioritizing the patient’s demands, which may result in neglect of the FC’s needs for support (25, 26). Consequently, despite the endeavors of healthcare professionals, the support requirements of family caregivers frequently go unaddressed (15, 21, 27). Nonetheless, the specific unmet support needs may differ significantly among FCs and fluctuate over time. Currently, the perspectives on preferred support and burden-related experiences, positive encounters, challenges, and assistance to carers of EoL patients remain ambiguous. Previous research on family caregivers’ assistance at the EoL has predominantly focused on certain disease categories (12, 20–22, 25, 28–30) or support alternatives (24, 31). Furthermore, assistance needs and experiences about the care situation are frequently evaluated by a quantitative methodology, predominantly yielding a numerical representation of the majority’s support preferences and experiences. Nonetheless, most perspectives fail to represent the diversity in support requirements and experiences among FCs. In turn, the disparities among FCs regarding their specific assistance needs and caregiving experiences remain overlooked. In Portugal, few studies have utilized a mixed-method approach, incorporating both quantitative and qualitative approaches, to analyse this complex phenomenon from a multidimensional stance. Mixed methods research is an advantageous technique that helps strengthen the evidence in PC and EoL research. As previously stated, FCs may experience an escalating burden that jeopardizes their physical and psychological well-being, hence impairing their caregiving capabilities. Comprehending the methods to mitigate these stressors is essential for fulfilling the growing requirements for home-based care. Primary care personnel require assistance in recognizing at-risk caregivers, providing them with support and thereby preventing unnecessary hospital stays for patients. Given the growing older population, routinely evaluating caregivers’ demands is essential to identify crisis situations and notify personnel of the escalating demands on carers (32). Considering the increasing demands on community health and private agency personnel, along with the financial ramifications of prolonged examinations, any screening instrument must be straightforward to administer, concise, and, crucially, encompass priority areas for regular evaluation with FCs. Therefore, screening and triaging based on needs should be an essential component of the assessment process. Former research has highlighted the global changes in where people die, shifting toward death in the community, in line with patients’ preferences (33). In contrast, the Portuguese reality reveals a tendency to die in hospitals and lack of community PC and FCs support, a model that might not be sustainable in the future (33). Despite the extensive international evidence on the demands of caregivers, from both the caregiver and professional viewpoints (34–36), so far there is no known research from Portugal investigating the prioritization of caregiver needs in the context of EoL care from these perspectives.

Therefore, the overall aim of this study is to comprehensively assess the unmet needs of FCs in home-based PC settings and their experiences interacting with PC services, and then propose strategies and recommendations for FC advocacy in PC and foster the design of person-centered supportive interventions. The specific objectives are to:

a) characterize unmet needs of FCs of palliative patients who are cared for at home, tracking care needs, health literacy levels, social support, FC burden and risk of prolonged grief;
b) explore the profile of Portuguese FCs who care for palliative patients in a home setting, distinguishing their support needs and experiences with caregiving;
c) understand the role, impact, and support of FCs of palliative patients when interacting with PC services;
d) identify the priority indicators for inclusion in an alert assessment tool for FCs who are caring for a person who is dying at home;
e) synthesize the evidenced-based recommendations for FC advocacy in PC.

## 2 Methods

### 2.1 Study design

This study will employ a multi-stage mixed-methods design (37). This methodology entails the concurrent gathering of quantitative and qualitative data within a singular investigation. This approach was chosen as it gathers diverse data to comprehend participant experiences with home-based PC. The quantitative aspect comprises a cross-sectional survey, while the qualitative part uses a descriptive qualitative method with open-ended questions to elucidate FC experiences and gather expert opinion through the Delphi method. Data integration through embedding will occur when data collection and analysis are linked at multiple points. Data from phases I and II will be used to develop a CAT measurement tool in phase III.

### 2.2 Study development

**Figure 1:**
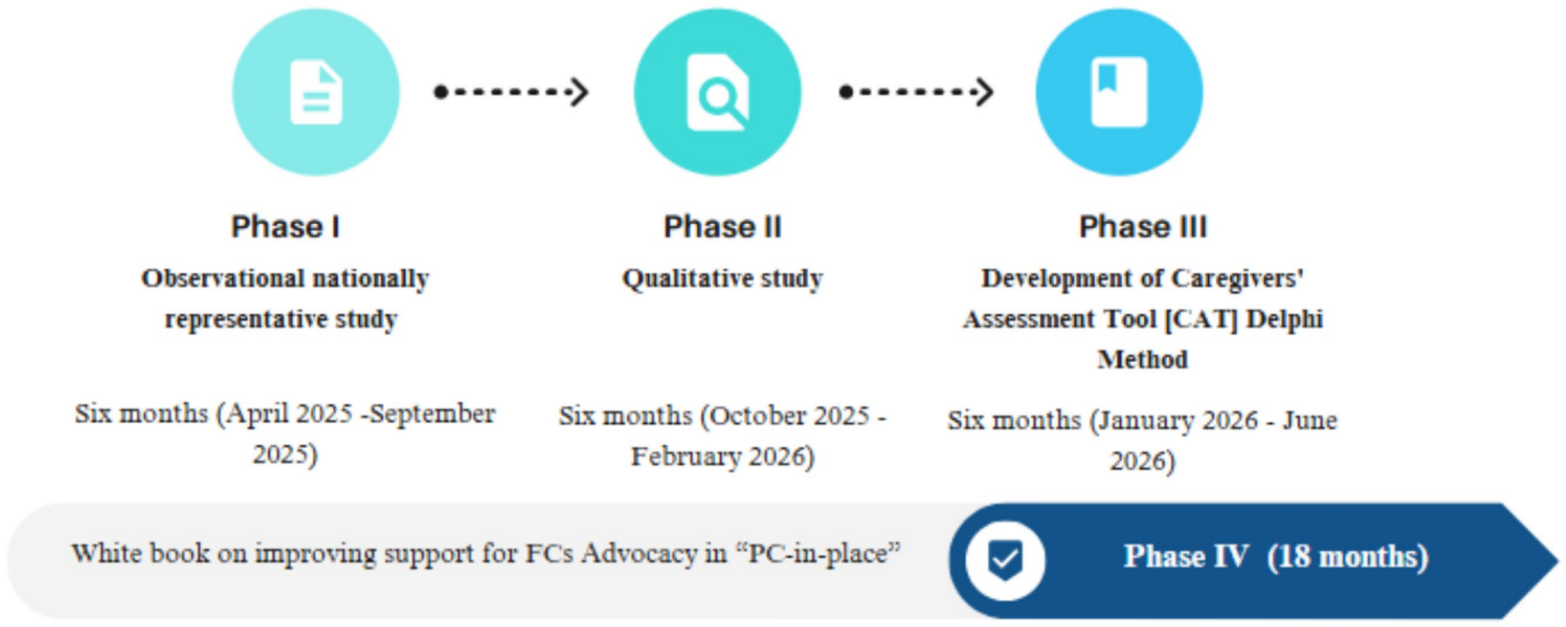
Overview of the study phases.

This study will be conducted in four major phases over a period of one and a half years, commencing from the date of execution in Portugal (Table 1). The research team possesses expertise in both qualitative and quantitative methodologies and holds graduate degrees in nursing (CL, AQ, and MD), psychology (AC), social work (CR), and medicine (RC). The four phases are (I) assessing FC needs and profiles; (II) assessing interactions with and access to PC services; (III) developing Caregivers’ Assessment Tool; and (IV) producing white book on improving support for FCs advocacy in “PC-in-place” (see Figure 1).

**Table 1:** Brief description of each study phase.

Phases	Main goals	Methods	Participants
Phase I	Characterize the unmet needs of FCs of palliative patients who are cared for at home, tracking care needs, health literacy levels, social support, FC burden and risk of prolonged grief Explore the profile of Portuguese FCs who care for palliative patients in a home setting, distinguishing their support needs and experiences with caregiving	Observational cross-sectional study	At least 500 FCs
Phase II	Understand the role, impact, and support of FCs of palliative patients when interacting with PC services	Qualitative study	50 FCs should achieve data saturation
Phase III	Identify the priority indicators for inclusion in an alert assessment tool for FCs who are caring for a person who is dying at home	Multi-phase modified Delphi method	**Round 1:** Qualitative Data Participants: 50 FCs **Round 2:** Delphi survey Participants: 40 PC professionals and 40 FCs **Round 3:** Expert panel Participants: Professionals with a strategic role in PC support within national or regional organizations (*n* = 10) and FCs (*n* = 10)
Phase IV	Synthesize the evidenced-based recommendations for FC advocacy in PC	Narrative-based approach and evidence review	Not applicable

#### 2.2.1 Phase I—observational nationally representative study (FCs’ needs and profiles)

This study will use a cross-sectional survey through telephone and in-person interviews with a large, nationally representative sample, to capture the unmet needs of users and to identify FC profiles. Inclusion criteria for the sample will be as follows: being primary FC; receiving home-based PC services; being ≥18 years old; and understanding Portuguese. FCs will be excluded if they have cognitive impairments that compromise their participation. Recruitment will be conducted according to the service list of the community support teams in PC from the Portuguese Observatory of PC. The surveys will utilize a random stratified sample of palliative family caregivers, categorized by gender, age range, and geographical area. The Strategic Development Plan for PC (2023–2024 biennium) estimates that 100,000 individuals require PC; they are supported by 64 community teams with regional coverage (38). Before data collection, *a priori* power analysis was performed using G*Power version 3.1.9.7 to ascertain the minimal sample size necessary for identifying a medium effect size (*r* = 0.30) with a two-tailed test, an α of 0.05, and a power of 0.95. This analysis revealed that at least 500 FCs are required for this study. Data will be gathered utilizing a structured questionnaire comprising six sections: (I) Sociodemographic details of the caregiver and the health status of the care recipient, including (i) the Edmonton Symptom Assessment Scale-revised to evaluate the severity of nine common symptoms in PC patients (39); and (ii) the Performance Palliative Scale (PPS) to estimate the survival duration of PC patients (40); (II) Health literacy levels of carers (41); (III) “Family Inventory of Needs” to measure the importance of care needs of families (42); (IV) Multidimensional Scale of Perceived Support to measure the perceived adequacy of social support from three sources: family, friends and significant others (43); (V) Caregiver Burden Scale to evaluate both the objective and subjective burdens of informal caregiving, gathering data on health, social life, personal life, financial status, emotional well-being, and the nature of the caregiver’s relationship (44); (VI) *Marwit Meuser* Caregiver Grief Inventory Short-Form to evaluate pre-death grieving (45). In terms of data analysis, we will utilize descriptive, inferential, and predictive statistics to summarize data, test hypotheses and forecast future outcomes, respectively. Data will be analyzed utilizing IBM SPSS Statistics version 29.0. The STROBE checklist (46) will be utilized.

#### 2.2.2 Phase II—qualitative study (interaction and access to PC services)

This qualitative study will explore the role, impact, and support of FCs of PC patients when interacting with PC providers. Furthermore, it is essential to identify the most effective methods for utilizing patient-level data in the creation and provision of PC. Caregiver participants will be purposively sampled (*n* = 50 should achieve data saturation) for face-to-face interviews. Inclusion criteria will be: (a) being primary FC; (b) receiving home-based PC services; (c) being ≥18 years old; and, (d) understanding Portuguese. Participants from the cross-sectional survey (phase 1) who provide consent will be asked to engage in a qualitative interview. Topic guides will focus on the experience of caregiving for individuals with palliative needs, existing engagement with and access to PC services, and expected clinical responses from health services, whether through in-person communication or digital technology. All interviews will be stored and analyzed via WebQDA software. A thematic analysis will be performed to delineate the conceptual framework of the principal topics derived from the interviews (47).

Some strategies will be implemented to guarantee qualitative rigor. Firstly, researcher reflexivity will be addressed throughout the research process by composing a reflective document on the researcher’s positionality that will be revisited at each study phase. Secondly, WebQDA software enhances transparency in analysis by allowing summaries or interpretations to be readily connected to the raw data. Thirdly, data from other sources will be compared to ascertain whether divergent findings emerge. Ultimately, a “thick description” will be incorporated into the final report, featuring instances of raw data (i.e., direct quotations from study participants) alongside pertinent contextual information regarding the participants (48). The research will adhere to the COREQ checklist (49).

#### 2.2.3 Phase III—development of caregivers’ assessment tool

This phase will design an assessment instrument to ascertain caregivers’ needs during routine practice by identifying and reaching consensus on the critical areas of need. Results from phases I and II will guide the creation of an initial Caregivers’ Assessment Tool (CAT), which will be enhanced through a multi-phase modified Delphi method (50) to achieve consensus among caregivers and professionals on a prioritized list of caregiver needs. This will inform the development of an assessment tool designed for the regular evaluation of caregiver needs, while addressing practical considerations for its practical implementation. A purposive sampling technique will be employed to recruit participants at each stage of the study, consisting of existing or bereaved caregivers, as well as professionals experienced in helping caregivers throughout the study phases. All volunteers must be at least 18 years old and capable of providing consent to participate in the study. Participant experiences are crucial to guarantee a diverse array of opinions on the primary demands impacting caregivers delivering end-of-life care to those dying at home. The initial phase (R1) will concentrate on generating items for the survey, utilizing thematic analysis of data obtained from semi-structured interviews and a focus group involving a target sample of 50 adult family caregivers selected from participants of phase 1. The outcomes from phases I and II will facilitate item production. The second round (R2) will employ a systematic method to analyse data gathered from online surveys with PC professionals and FCs. Round 2 survey will comprise 40 professionals and 40 caregivers (*n* = 80). A third round (R3) will analyse data gathered from two groups: professionals with a strategic role in PC support within national or regional organizations (*n* = 10) and FCs (*n* = 10). This data will be utilized to further refine the final elements for inclusion in the CAT. We will employ triangulation methods to synthesize qualitative and quantitative data (51). Findings will be integrated into feedback reports, emphasizing areas of agreement and disagreement among participants. To ensure thorough reporting, we will synchronize qualitative codes with quantitative items, authenticate quantitative themes through qualitative data, and conduct contextual analysis to clarify the rationale behind quantitative ratings. We will also comprehensively document the triangulation process, including decision-making rationales and integration procedures, to enhance trustworthiness.

#### 2.2.4 Phase IV—white book on improving support for FCs advocacy in “PC-in-place”

A White Book will be developed to synthesize evidence-based suggestions for FCs Advocacy in “PC-in-place,” drawing on outcomes from prior assignments and a vast research literature. This book will assess the contributions of FCs to home-based care and elucidate the significance of their work as a crucial resource in PC. We will delineate the problems confronting the FCs of patients with primary care needs and propose best strategies for service providers, stakeholders, and decision-makers to collaborate with these carers.

## 3 Discussion

A critical policy issue pertains to the establishment of egalitarian societies, wherein robustly supported access to healthcare and social systems empowers individuals and communities to actively participate in their own development and influence the 2030 Agenda through local, national, and global actions. The paucity of research on effective home-based PC has limited our ability to identify the most effective avenues for future policy and practice. To ensure QoL for palliative patients and their caregivers, compassionate-friendly care services are needed (52). By offering novel data on FC needs and the critical aspects faced when caring, the project will yield important insights about how to foster a more inclusive society. Consequently, new strategies should be developed to comprehensively assess the unmet care needs of FCs before designing and providing tailored PC services, to ensure QoL in PC.

The project’s outcomes will enrich the community’s PC while optimizing social welfare procedures. Moreover, by identifying the unmet requirements of FCs, the initiative will enhance the QoL and well-being of care recipients. Addressing the needs of FCs should improve their health and competence to care, minimizing the consumption of hospital health resources, while attending to patient preferences. Current understanding of significant FC engagement will be harmonized and sustainably executed through the participation of pertinent stakeholders. The project will provide an innovative network platform and essential resources to enhance the present welfare system response to the requirements of palliative patients and their FCs. Furthermore, it will include knowledge-driven suggestions for FC advocacy, guidance on FC educational interventions, accessible resources to be mobilized in the territory, and strategies to streamline and integrate the caregiving process. This will also diminish the waste of health and social resources. Finally, this study could be the first step in the development of a “PC-in-place” barometer that responds to concerns regarding the culture of political correctness in the practice setting and its influence on the experiences of patients, family caregivers, and staff. It will also facilitate a deeper exploration and encourage discourse around personnel matters, particularly at the team level.

The study’s main asset is that it will be the first research undertaken at the national level with representative sampling. To our knowledge, no prior research has utilized such rigorous methods, especially targeting family caregivers—a frequently neglected group. A drawback of this study is that its cross-sectional design precludes the establishment of causation regarding the relationships. Furthermore, additional environments such as nursing homes will fall outside the study scope, constraining the findings’ representativeness and transferability. Another possible limitation of the research is that it might not be applicable to locations outside of Portugal. Considering the possible impact of location-specific elements is vital when evaluating and applying the study’s conclusions. In addition, to make the most of the study’s relevance and applicability, future research should investigate how to adapt and use the findings in other contexts and cultures. Another anticipated limitation is that interview-based research relies on self-reported actions and attitudes, which can be influenced by recall and social desirability biases. To reduce social desirability bias, we will assure participants that their data will remain anonymous. Lastly, potential selection bias or logistical challenges in conducting face-to-face interviews could affect the validity and reliability of the collected data.

## 4 Ethics and dissemination

This study received ethical approval from the Research Ethics Committee of the Polytechnic University of Leiria (CE/IPLEIRIA/11/2025). Any substantial changes to the ongoing protocol will be submitted as an amendment for approval. All research members agree to adhere to the Guidelines for the Responsible Conduct of Practice according to the Declaration of Helsinki. Participants will be assured that all information will be kept secure and anonymous, that participation is voluntary, and that it may be retracted at any time without repercussions. Informed consent will be acquired at every stage. Volunteers will receive no compensation for their participation. Coded information will be employed in data management to provide privacy protection. Data will be preserved safely and confidentially, in compliance with institutional laws for data storage. The consent forms, data collection forms, and full transcripts will be preserved for a period of 5 years.

The key findings will be communicated via pertinent social media platforms, websites, and succinct reports to relevant entities and stakeholders. Scholarly articles will be submitted to relevant peer-reviewed journals. Additionally, public presentations will be presented at relevant national and international conferences.

## 5 Conclusion

This study protocol delineates strategies for collecting ample information on the needs of FCs, thereby addressing shortfalls in PC research. The study findings will provide policymakers and stakeholders with essential insights into family caregiving issues that should be prioritized in Portugal’s current strategic plan for PC development. The project has the potential to empower FCs to become relevant key stakeholders in their communities. Furthermore, it will offer knowledge-based suggestions for FC advocacy, guidance on FC educational interventions, accessible resources to be mobilized at the country level, and enhance the caregiving process to be more cohesive and less disjointed. By producing a White Book on improving support for FCs advocacy in “PC-in-place,” the project can target different publics to improve support for family carers in PC.
